# Supplementary material for: Deviance detection and regularity sensitivity in dissociated neuronal cultures
Source: Front Neural Circuits. 2025 Aug 25;19:1584322. doi: 10.3389/fncir.2025.1584322 (PMC12415055; doi:10.3389/fncir.2025.1584322)
Supplement: Supplementary file 1 [file Data_Sheet_1.PDF]

Supplementary Information for

Deviance Detection and Regularity Sensitivity in Dissociated Neuronal Cultures

Zhuo Zhang, Amit Yaron, Dai Akita, Tomoyo Isoguchi Shiramatsu, Zenas C. Chao, Hirokazu Takahashi

Correspondence to: Hirokazu Takahashi

Corresponding author. Email: takahashi@i.u-tokyo.ac.jp

This PDF file includes:

Supplementary Figure 1 to 3

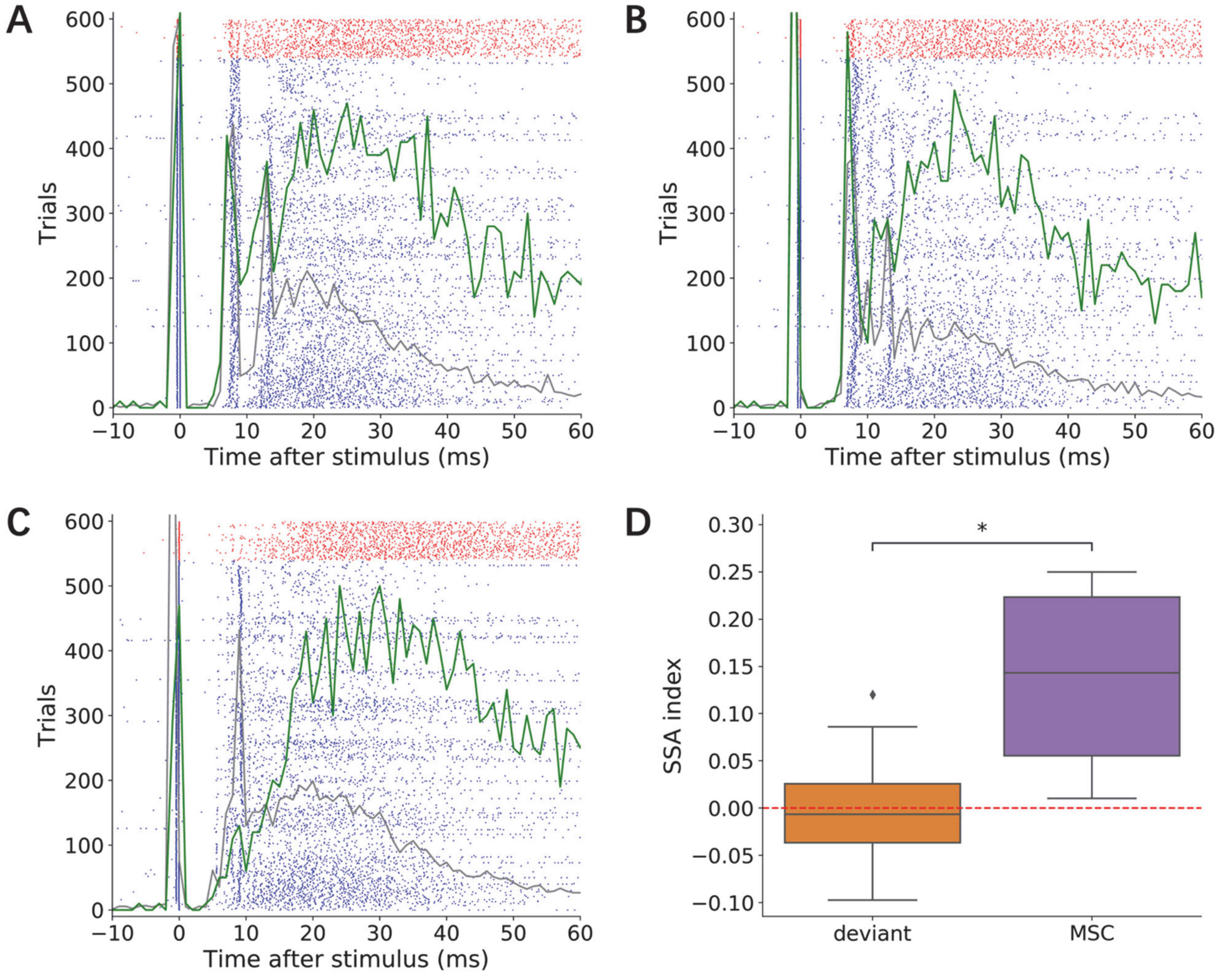

Supplementary Figure 1. Early responses. (A) Example of neural responses where the early response to the standard stimulus is equal to the deviant response. (B) Example showing a smaller early response to the standard stimulus compared to the deviant response. (C) Example displaying a greater early response to the standard stimulus than that to the deviant response. The raster plots depict neuronal responses to standard (blue dots) and deviant (red dots) stimuli recorded from a single electrode channel. The lines represent the averaged responses for standard (gray) and deviant (green) stimuli. (D) SSA index of early-phase responses (0–10 ms).

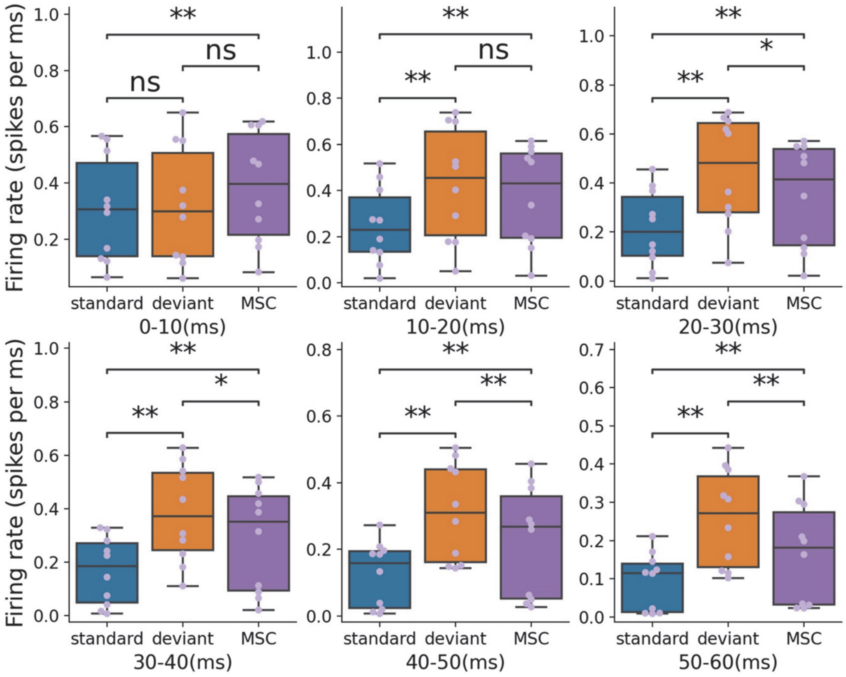

Supplementary Figure 2. Deviance detection in late responses. The box plots showed the responses to stimulus in different time windows at 0-10 ms, 15-30 ms, 30-40ms, 40-50 ms, and 50-60 ms. SSA was observed at the first 10ms, while deviant detection after 30ms.

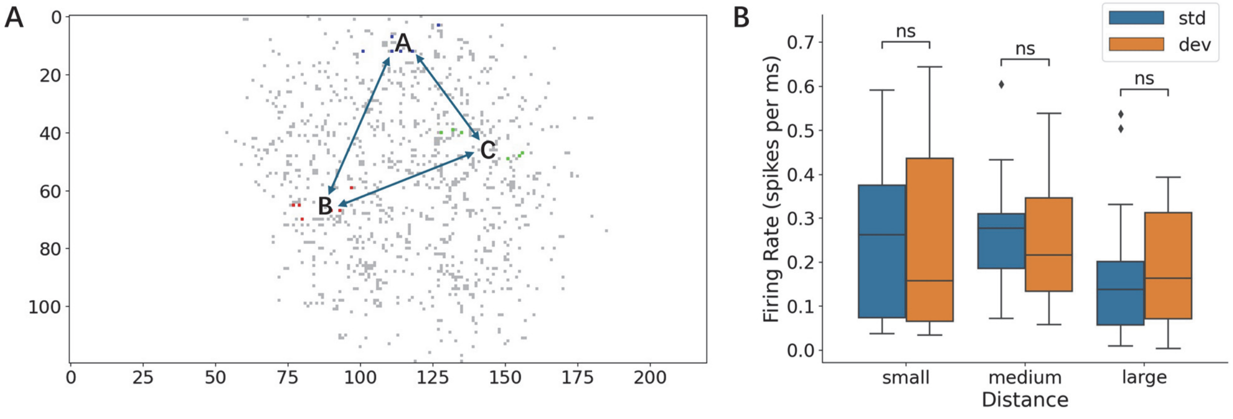

Supplementary Figure 3. Distance between stimulation electrodes did not affect early-phase responses. (A) Example of the distance between standard and deviant stimulation electrodes. (B) Early responses to standard and deviant stimuli as a function of the distance between standard and deviant stimulation electrodes.
